# Supplementary material for: An Unusual Resurgence of Human Metapneumovirus in Western Australia Following the Reduction of Non-Pharmaceutical Interventions to Prevent SARS-CoV-2 Transmission
Source: Viruses. 2022 Sep 28;14(10):2135. doi: 10.3390/v14102135 (PMC9612024; doi:10.3390/v14102135)

Supplementary material

Supplementary Table S1: Comparison of hMPV incidence per 100,000 per population group (with 95% CI) in Western Australia by calendar year between 2017 to 2021.

	Year				
	**2017**	**2018**	**2019**	**2020**	**2021**
All	19.1 (17.5-20.8)	23.5 (21.7-25.4)	28.9 (26.9-31)	6.8 (5.9-7.9)	70.6 (67.5-73.8)
Region					
Metropolitan	17.2 (15.4-19)	19.2 (17.4-21.2)	25.5 (23.4-27.8)	3.9 (3.2-4.9)	47.4 (44.5-50.4)
Northern	61.3 (47.2-79.7)	64.6 (50.1-83.4)	73.4 (57.8-93.2)	81 (64.5-101.8)	388.7 (350.4-431.3)
Southern	19.2 (15.9-23.3)	32.9 (28.4-38.1)	34 (29.4-39.3)	5.2 (3.6-7.5)	104 (95.8-113)
Age-group					
<12 months	239.7 (192.3-298.7)	288.2 (235.8-352.3)	294.3 (241.3-359)	54.6 (34.4-86.7)	685.7 (602.1-780.8)
1-4 years	77.2 (63.9-93.4)	102.5 (87-120.8)	95.3 (80.4-113)	16.6 (11-25)	384.8 (353.5-418.8)
5-15 years	7.2 (4.9-10.5)	13.5 (10.3-17.8)	17.8 (14-22.6)	3.7 (2.2-6.3)	48.3 (41.2-55.9)
16-64 years	9.1 (7.8-10.6)	11.1 (9.6-12.8)	13.4 (11.8-15.3)	5.5 (4.5-6.7)	35.2 (32.5-38.2)
≥65 years	34.4 (29.1-40.6)	36.6 (31.2-42.9)	59.9 (52.8-67.9)	8.3 (6-11.7)	92.5 (83.7-102.4)

Supplementary figure S1: The Moving Epidemic Method R graphical output of hMPV detections between 2013 and 2019, including year to year comparison of seasons onset and offset thresholds. Panel A depicts the weekly rate, epidemic threshold and season duration. Panel B demonstrates the season onset and offset and intensity thresholds.

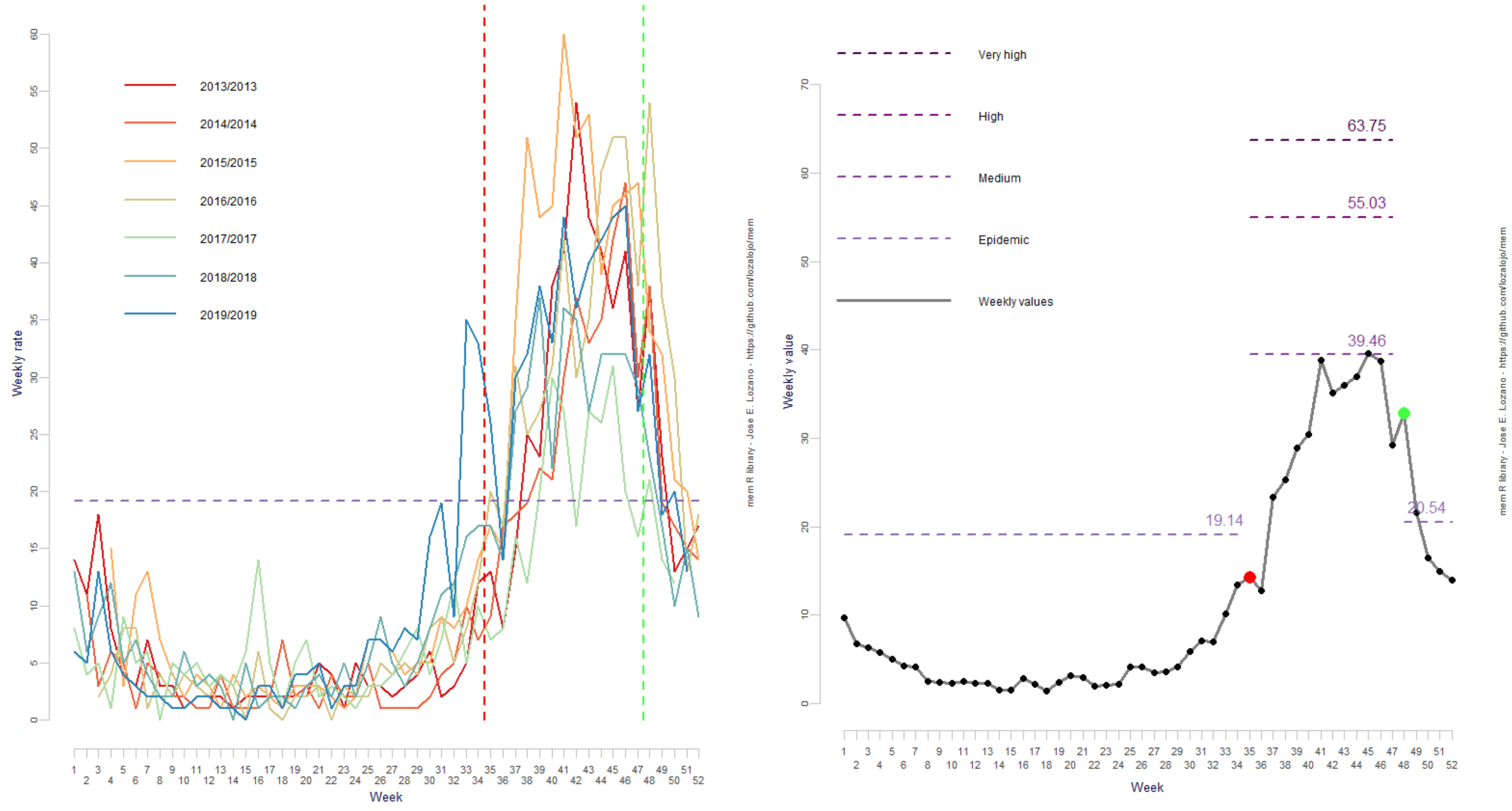

Supplementary figure S2 Panel A-C: hMPV tests and detections per week by region between week one, 2017 and week 52, 2021. A) Detections and tests per week in the Metropolitan region. B) Detections and tests per week in the Northern region. C) Detections and tests per week in the Southern region. The average epidemic curve is based on all hMPV detections in PathWest Laboratory Medicine between 2013 and 2019, inclusive. The first local case of SARS-CoV-2 was in week eight of 2020. State-wide stay at home order (weeks 14-17 of 2020) was followed by sequential lifting of restrictions (weeks 18-26) with gatherings of 10 allowed at week 18, 20 at 21 weeks and 100 at week 24. Periods of increased NPIs included mask requirements in Metropolitan and Southern regions (weeks 5-6, weeks 16-19 and 26 - 27) in 2021. hMPV, human metapneumovirus; NPI, non-pharmaceutical intervention

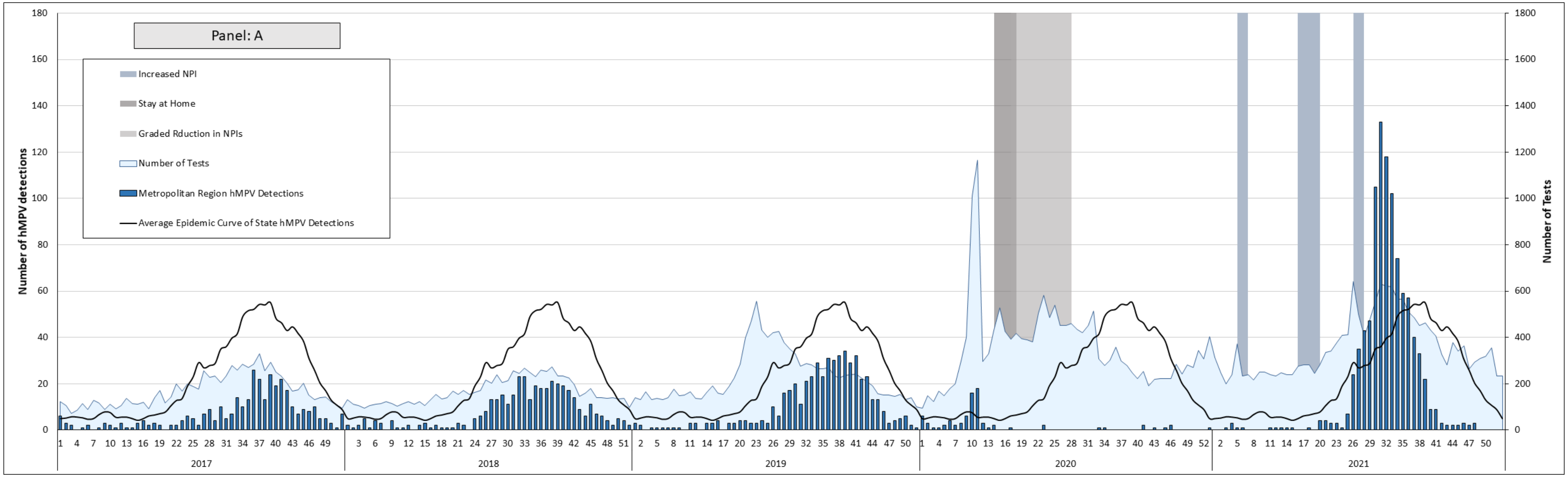

(A)

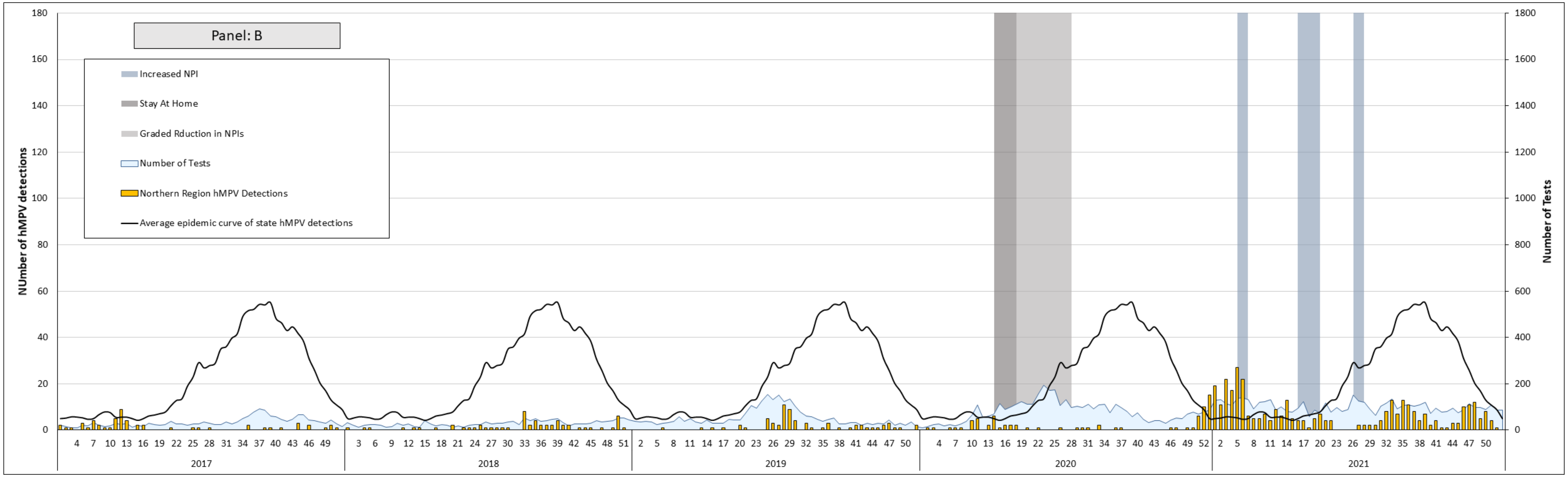
Panel: B
Increased NPI
Stay At Home
Graded Rduction in NPIs
Number of Tests
Northern Region hMPV Detections
Average epidemic curve of state hMPV detections
NUmber of hMPV detections
Number of Tests
2017
2018
2019
2020
2021

(B)

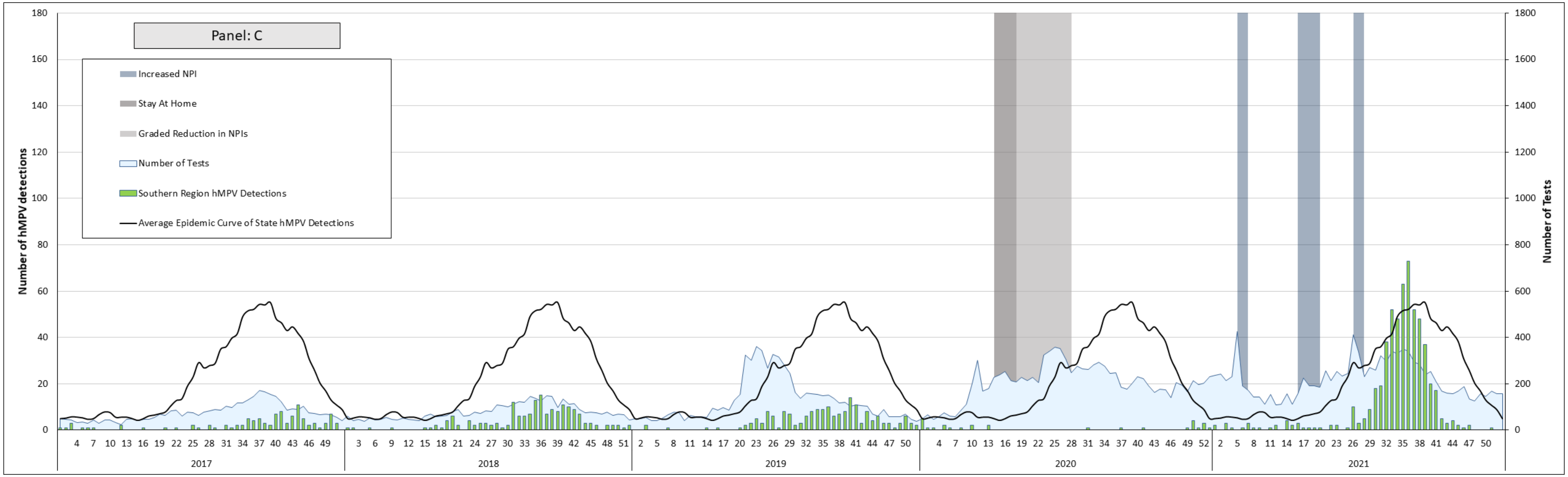
Panel: C
Increased NPI
Stay At Home
Graded Reduction in NPIs
Number of Tests
Southern Region hMPV Detections
Average Epidemic Curve of State hMPV Detections
Number of hMPV detections
Number of Tests
2017
2018
2019
2020
2021

(C)

Supplementary figure S3: hMPV detections per week by age group, between 2017 and 2019, inclusive, and 2021. The determined hMPV season for each year is demarcated with a shaded region. hMPV, human metapneumovirus

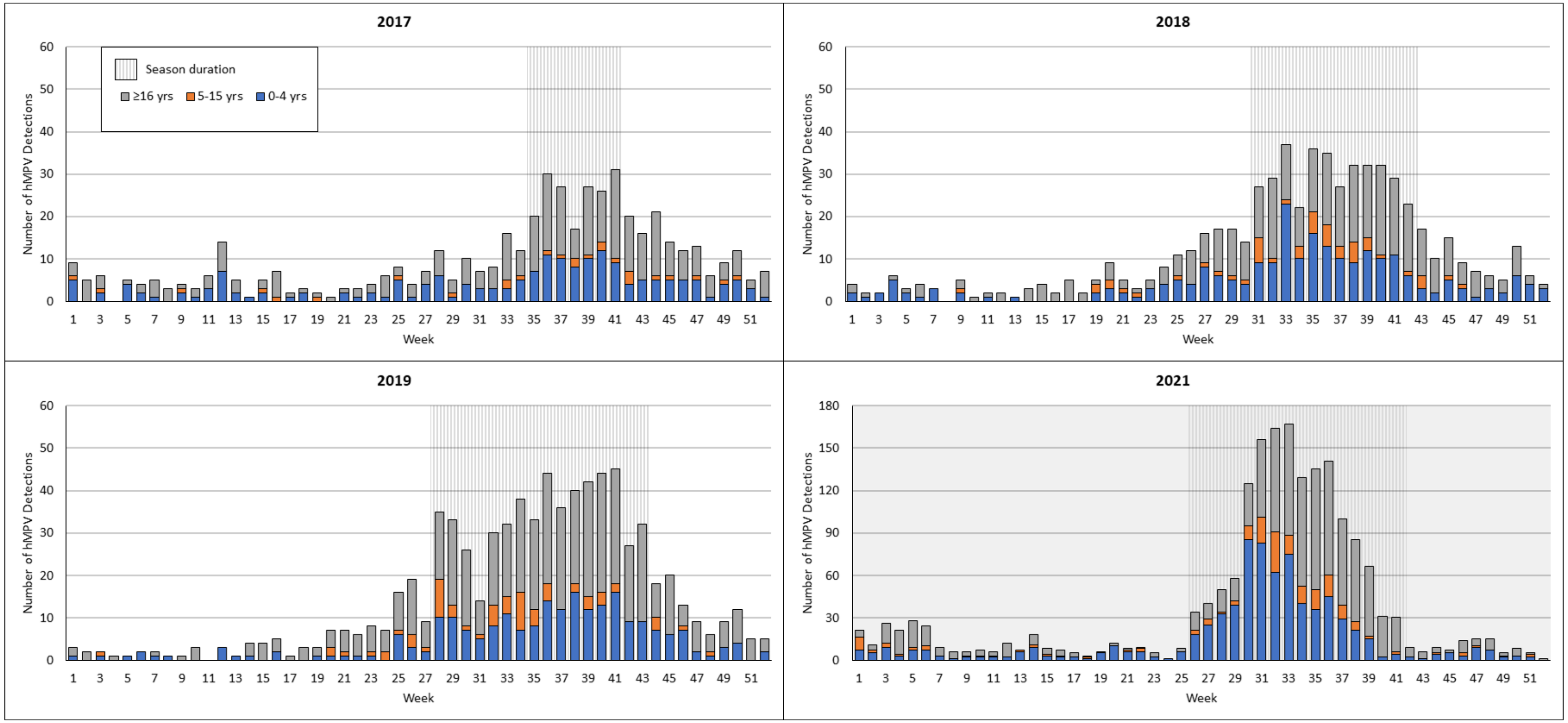

Supplementary figure S4: Phylogenetic analysis of hMPV lineage B viruses sampled in Western Australia (WA) between 2017 and 2020. WA hMPV genomes sampled during the period are shaded according to the year of sampling. Genomes sampled in 2017 are shaded purple, genomes sampled in 2018 are shaded red, genomes sampled in 2019 are shaded blue and genomes sampled in 2020 are shaded green. Reference sequences downloaded from GenBank are shaded pale orange. The scale represents the number of nucleotide substitutions per site.

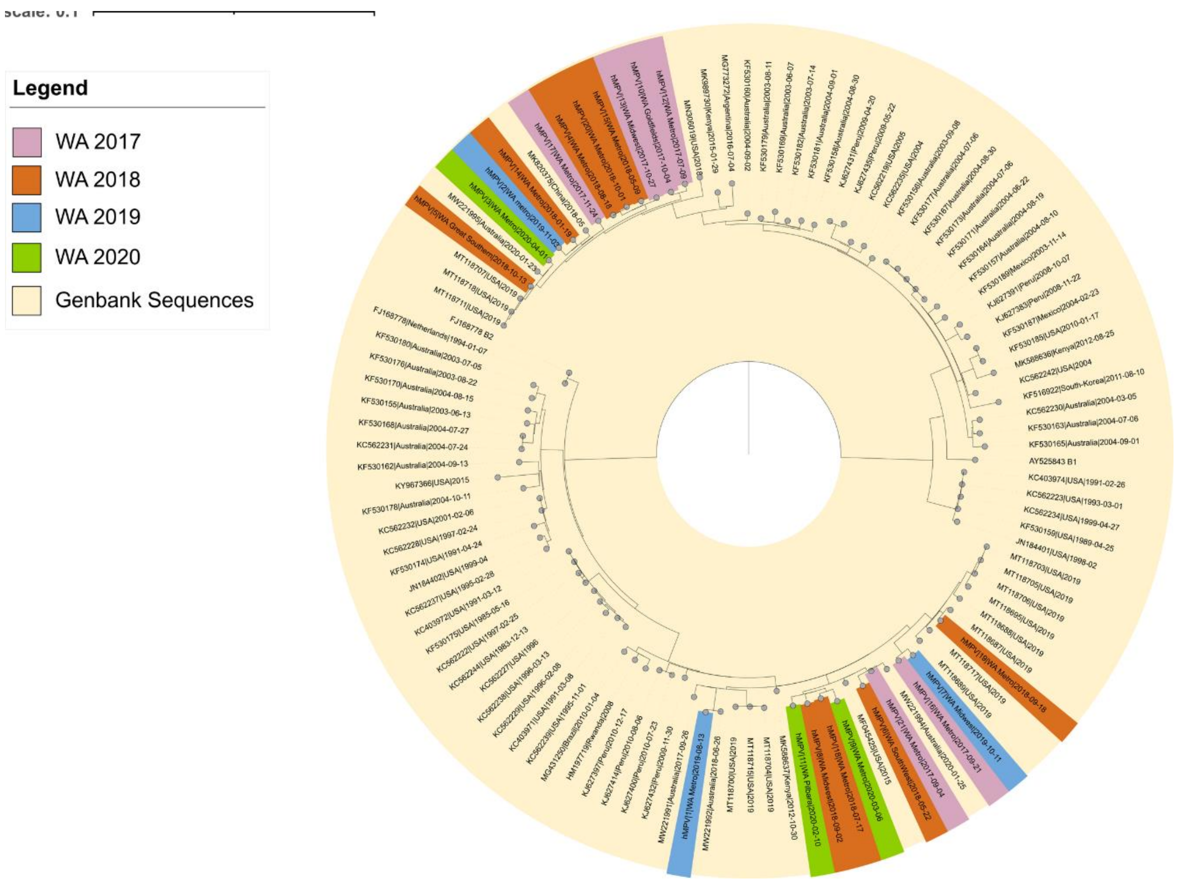

Supplement: Supplementary file 1 [file viruses-14-02135-s001.zip › viruses-1915796-supplementary.pdf]
